# The Effect of CO_2_ Laser Irradiation Combined with TiF_4_ and NaF Varnishes on Enamel Hardness: An In Vitro Study

**DOI:** 10.3290/j.ohpd.a44690

**Published:** 2020-07-04

**Authors:** Hajar Dehghan, Farzad Mojarad, Maryam Serajzadeh, Reza Fekrazad

**Affiliations:** a Pedodontist in Private Practice, Tehran, Iran.; b Associate Professor, Paediatric Dentistry Department, Dental Faculty, Hamadan University of Medical Sciences, Hamadan, Iran.; c Dentist in Private Practice, Tehran, Iran.; d Professor of Radiation Sciences Research Center, Laser Research Center in Medical Sciences, AJA University of Medical Sciences, Tehran, Iran; International Network for Photo Medicine and Photo Dynamic Therapy (INPMPDT), Universal Scientific Education and Research Network (USERN), Tehran, Iran.

**Keywords:** CO_2_ laser, enamel hardness, TiF_4_ varnish

## Abstract

**Purpose:**

To assess the effects of experimental titanium tetrafluoride (TiF_4_) varnish and commercial sodium fluoride (NaF) varnish with CO_2_ laser on enamel hardness.

**Materials and Methods:**

Ninety human enamel samples were randomly assigned to one of the following groups: 1. control (no treatment) (CO); 2. NaF varnish (2.26%) (NF); 3. TiF_4_ varnish (2.45%) (TF); 4. CO_2_ laser (La); 5. NaF varnish (2.26%) with CO_2_ laser (NFL); 6. TiF_4_ varnish (2.45%) with CO_2_ laser (TFL). Enamel surface changes were determined by Vickers microhardness (VH) test with a load of 1000 g and a dwell time of 12 s. Each sample was indented three times. Data were analysed using one-way ANOVA and Tukey’s test.

**Results:**

The mean surface microhardness was 245.5 VH in the CO group, 280.3 VH in group NF, 338.7 VH group TF, 277.0 VH in group La, 345.3 VH in group NFL, and 368.0 VH in group TFL. Statistical analysis showed that groups TF, NFL, and TFL had statistically significantly higher surface hardness than the control group (p < 0.05).

**Conclusion:**

The microhardness of enamel treated with TiF_4_ varnish with or without laser irradiation was statistically significantly greater than that of the control group. Thus, using TiF_4_ to increase enamel surface microhardness can be recommended.

Althouh the application of conventional fluorides has been effective in decreasing caries by 50%-60% in permanent teeth and 40%-50% in deciduous teeth, caries remains the most prevalent childhood chronic disease.^30^

Fluorides could potentially be used to prevent demineralisation, e.g. sodium fluoride (NaF), which is related to the formation of a calcium fluoride (CaF_2_) layer which acts as a physical barrier or as a mineral reservoir. However, the protective ability of sodium fluoride is limited, because the layer it creates is soluble in acids. Some studies have focused on other fluorides which contain polyvalent metal ions, such as titanium tetrafluoride (TiF_4_), which may be more effective in preventing demineralisation by forming an acid-resistant surface layer, increasing fluoride uptake, and incorporating titanium in hydroxyl apatite lattice.^1,23,24,26^ The application of TiF_4_ seems to increase fluoride uptake, reduce acid solubility, and increase penetration when compared with the application of NaF.^6,24,26^ Although much research has shown the efficacy of TiF_4_ in reducing enamel erosion,^23,25,26,32,41,44^ some studies found no protective effects associated with its application.^24,27^ According to findings, TiF_4_ is most effective in the form of a varnish vs a solution.^22,26^ Despite these findings, the most common varnish used in dentistry to prevent caries is sodium fluoride.^26^

Fluoride alone is more effective on smooth than pitted, fissured surfaces, such as the occlusal surface. But as research has shown, the preventive effect of CO_2_ laser on occlusal surfaces is similar to its effect on smooth surfaces.^34^ Therefore, according to some papers, combining laser irradiation and fluoride therapy increases the effects of different fluorides on enamel demineralisation.^14, 21,26,34,40^

Different methods involving laser irradiation have been used, such as laser-assisted fluoride therapy (LAFT), and laser combined with the application of fluoride, either prior to or following laser irradiation. It seems that the combined method is more effective. There are different suggestions for increasing enamel acid resistance after some types of laser irradiation, such as decreasing enamel permeability and chemical changes, or both.^30,38^

The results of several studies have shown that laser irradiation can decrease demineralisation inside the enamel. It has been suggested that the absorption of CO_2_ laser irradiation by enamel and dentin is greater than that of other kinds of laser. The CO_2_ laser appears to be a better choice compared with other types of laser, since it has greater surface absorbance and less penetration depth^.30,35.38^

The aim of this study was to evaluate the effects of CO_2_ laser irradiation on enamel microhardness after applying TiF_4_ varnish and NaF varnish.

## Materials and Methods

### Specimen Preparation

Fifteen human premolars extracted for orthodontic reasons were used in conformity with the rules of the Research and Ethics Committee of the Faculty of Dentistry, Hamedan University of Medical Sciences (Process No.13900431/64). The teeth were checked with a 10X lens and radiography to ensure they were free of caries, cracks, fillings, abrasions, or any enamel defects. The samples were kept in a 0.1% thymol solution at room temperature during specimen preparation.^28^

The teeth were cut with a cutting machine (DEMCO Nonstop E6-236: Oklanoma City, OK, USA) into 6 enamel specimens (3 x 3 x 2 mm) from each tooth. Each of the 90 enamel specimens was randomly allocated to one of the following groups: 1. control (untreated) group (CO); 2. NaF varnish (NF); 3. TiF_4_ varnish (TF); 4. CO_2_ laser (La); 5. CO_2_ laser and NaF varnish (NFL); 6. CO_2_ laser and TiF_4_ varnish (TFL). The samples were embedded in acrylic resin and ground flat with water-cooled carborundum disks (320-, 600-, and 1200-grit Al_2_O_3_ papers, Buehler; Lake Bluff, IL, USA) and then polished.

### Treatment

No treatment was performed on the control group. In groups NF and TF, NaF varnish (2.26% F, Duraphat, Colgate; Sao Paulo, Brazil) and TiF_4_ varnish (FGM-Pent Searle; Joinville, SC, Brazil 2.45%), respectively, were applied with a microbrush according to the manufacturers’ instructions. The La samples were irradiated with a CO_2_ laser (Smart US 20D, Deka: Florence, Italy) with a 10.6-µm wavelength, and 2 W power for 10 s in continuous wave (CW) mode at a distance of 10 mm from the enamel surface. Power density was 4 W/cm^2^ and total energy density was 40 J/cm^2^. The spot size was 8 mm, and irradiation was performed in a continuous scanning motion that allowed the entire surface to be irradiated. The specimens in group NFL and TFL were treated with NaF varnish and TiF_4_ varnish in the same manner as previously described, then immediately irradiated with the CO_2_ laser at the same specifications as in group La.

**Fig 1:**
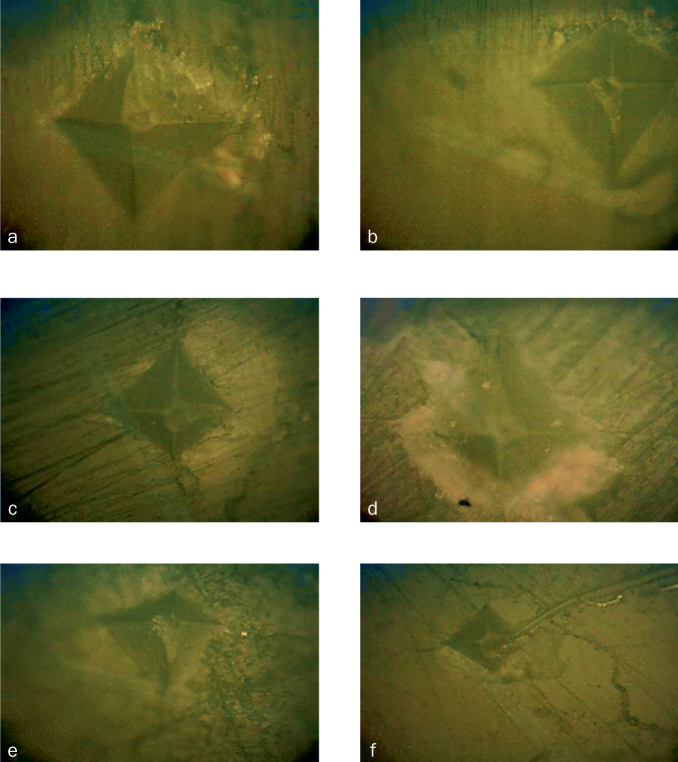
Microscopic views of each group after the indenting. a. CO = control group; b. NF = NaF varnish; c. TF = TiF_4_ varnish; d. La = CO_2_ laser; e. NFL = CO_2_ laser and NaF varnish; f. TFL = CO_2_ laser and TiF_4_ varnish.

Enamel microhardness was tested using the Vickers microhardness test (Digital Vickers, VMT, X series, Matsuzawa; Akita, Japan). Three indentations were made on each enamel surface using a load of 1000 grams, an application time of 12 s and a 150-µm distance between each position of the indenter. The minimum and the maximum values were omitted, with the middle number entered into the following formula to calculate the hardness: VHN = Fx1.85/d^2^, where VHN = Vickers Hardness Number, F = kg/m^2^, and d = mean diameter. The microscopic images are shown in Fig 1.

### Statistical Analysis

The one dependent variable was surface hardness of enamel. The independent variable was the method used to influence enamel surface hardness. There were six experimental groups. The findings were analysed statistically using SPSS 16 (IBM; Armonk, NY, USA). ANOVA followed by Tukey’s multiple comparison test were used to evaluate the statistical significance of all pairwise comparisons. The significance level was set at p < 0.05.

## Results

Mean enamel hardness was lowest in group CO (245.49) and highest in group TFL (367.96). The enamel hardness in group NF was 280.3, 338.75 in group TF, 277.02 in group La, and 345.33 in group NFL.

The mean enamel surface microhardness in different groups and their multiple comparisons are given in Tables 1 and 2.

**Table 1:** Mean enamel surface microhardness values in the different groups (n = 15) (p < 0.05)

Group	Sample size	Mean surface hardness	SD
Control	15	245.5	51.4
NaF	15	280.3	46.8
TiF_4_	15	338.7	95.5
CO_2_	15	277.0	66.3
NaF + CO_2_	15	345.3	57.7
TiF_4_ + CO_2_	15	368.0	112.3
Total	90	303.6	88.4

**Table 2:** Significant differences between groups (p-values) and multiple comparisons based on Tukey’s HSD*

Group	CO	NF	TF	La	NFL	TFL
CO		1.000	0.014***	0.862	0.007***	0.000***
NF			0.016***	0.886	0.008***	0.000***
TF				0.232	1.000	0.896
La					0.143	0.017***
NFL						0.963
TFL						

* HSD = Honestly Significant Difference. Control group = CO; NaF varnish = NF; TiF_4_ varnish = TF; CO_2_ laser = La; CO_2_ laser and NaF varnish = NFL; CO_2_ laser and TiF_4_ varnish = TFL. ***There is a statistically significant difference between the means of the two groups (p < 0.05).

## Discussion

Fluoride application is one of the most effective ways to inhibit caries. Fluoride increases the resistance of enamel to demineralisation by increasing remineralisation and changing the tooth structure.^12^ Recent studies have shown that the use of topical fluoride is more effective than using systemic fluoride in preventing caries.^2,5^

The present study examined the effects of CO_2_ laser with NaF and TiF_4_ varnishes on enamel surface hardness using laser-assisted fluoride therapy (LAFT) and the Vickers microhardness test. This study found that TiF_4_ varnish increased enamel surface hardness considerably. NaF varnish and CO_2_ laser alone had lesser effects; however, CO_2_ irradiation statistically significantly increased the effects of both NaF and TiF_4_ varnish (p < 0.05).

In this study, 2.45% TiF_4_ varnish and 2.26% NaF varnish were compared. These varnishes and these fluoride concentrations were chosen for their widespread clinical use and favorable effects proven in previous studies.^24,37^ The composition of the experimental TiF_4_ varnish is similar to that of the NaF varnish. Different investigations studied TiF_4_ in the form of a varnish or a solution.^23-27,42,43^ TiF_4_ varnish has the ability to adhere to the tooth surface, which allows increased contact time with the enamel, thereby prolonging the reaction between fluoride and the enamel surface, thus increasing the uptake.^22,43^

In several studies,^3,6,7,26^ comparisons of TiF_4_ and other fluoride products including NaF showed the superiority of TiF_4_, which corresponds with the results of the current study, although this study used TiF_4_ varnish, which is more effective than its solution form.^26^ The protective action of TiF_4_ is not due only to fluoride, but also to its titanium content, because the latter forms a TiO_2_ glaze, an organometallic complex of titanium and organic dental matrix, which is probably effective in making and increasing the hardness of the enamel surface.^3,9,39,43^ Although the mechanism of forming such a layer after the application of TiF_4_ has yet to be elucidated, it is possible that a new complex – hydrated titanium phosphate – is formed, which could explain the better results of these groups.^17^

Magalhaes et al^27^ showed that NaF varnish is effectively reduces enamel softening but that it has no effect on the reduction of wear; nor was the TiF_4_ varnish able to reduce enamel softening and wear. In another study by Magalhaes et al,^24^ there was no difference between the control group and the other groups containing TiF_4_ and NaF solutions and varnishes. The results of those two studies diverged from those of the present study, which may be because the Magalhaes studies used bovine root dentin. Previous studies have shown that bovine teeth have different susceptibility and reactions to acid and TiF_4_ compared to human teeth.^17,33^ On the other hand, dentin has a lower mineral content than enamel and is more susceptible to erosion. In the Magalhaes et al study,^24^ a large amount of the mineral content of dentin was lost in the first erosive cycle, and the organic content was exposed. This might be the reason for the lower efficacy of fluoride products in that study.

CO_2_ laser was used in the present research to assess its effect on the application of topical fluoride based on recommendations of previous investigations and the high surface absorbance.^10,35^ Transformations in the crystalline phase, changes in chemical composition, and a reduction in acid permeability due to surface alterations (such as fusion and the melting of the crystallites) result from laser therapy.^35,38^ CO_2_ laser application could be effective in increasing fluoride deposits on enamel surfaces. These precipitates have weak attachments to dental surfaces, act as reservoirs for fluoride, and are released when needed.^34,35^ The CO_2_ laser could also increase fluoroapatite crystal formation with strong bonds to the crystal structure of dental tissue.^29,34^ Both forms of fluoride, superficial and fluoroapatite crystals, can be released from dental structures during an acid attack and induce remineralisation and enamel surface hardness.^18,19^ Applying varnish before laser therapy may create a mechanical barrier to laser irradiation.^26^ Since laser irradiation and fluoride therapy were applied simultaneously in this study (LAFT), it seems that the possible mechanism was only the thermal effect of the laser, with the laser irradiation working as an accelerator for the fluoride interaction. Laser irradiation has little effect in terms of changing the surface structure, since it is not in direct contact with the enamel surface, and the fluoride varnishes act as barriers.^30^

SEM observations by Magalhaes et al^26^ did not show any changes in the enamel structure after the applications of TiF_4_ and laser therapy. It seems that the better results of simultaneous application of fluoride and laser are due to the increase in temperature and the increased reaction between TiF_4_ and hydroxyapatite.

Different studies have used the combination of fluoride and laser irradiation with different kinds of laser and fluoride. Most of them show that fluoride and laser irradiation mutually enhance their effects.^3,4,8,11,14,16,19,21,26,34,35^ However, some other studies found no significant differences in reduction of enamel permeability and increased enamel microhardness between the groups using TiF_4_ alone or TiF_4_ preceding CO_2_ laser irradiation.^13,19^

Tepper et al^38^ found that CO_2_ laser irradiation assisted by fluoride solution increased the acid resistance of enamel specimens. Those authors used a continuous-wave CO_2_ laser with 2 W power and 10.6 µm wavelength for 15 s simultaneously with fluoride. The same parameters were used in the present study, with the exception that Tepper et al’s study found no significant difference between groups, except compared to the control group. Rodrigues et al^34^ also found that CO_2_ laser inhibits enamel demineralisation, and when accompanied by fluoride, its effect increased. Those authors suggested that the laser is more effective than fluoride in preventing caries, possibly because they used fluoride-containing toothpastes which have a lower concentration of fluoride and would therefore have lower results than professional fluorides with higher concentrations.^15,28^ Nemati et al^30^ also investigated the effects of two kinds of laser – CO_2_ and Er,Cr:YSGG – assisted by acidulated phosphate fluoride (APF) (LAFT) on enamel demineralisation and discovered that all the techniques used in controling caries were effective with no statistically significant differences from the control group. However, the combination of CO_2_ laser (10.6 µm, peak power of 291 W, for 10 s) and APF yielded significantly better results than the other groups.

Wiegand et al^43^ explored the effects of TiF_4_ and amine fluoride together with CO_2_ laser on enamel and dentin abrasion and found that amine fluoride solution is more effective than TiF_4_. However, CO_2_ laser statistically significantly increased the effect of TiF_4_.

After studying the effect of varnish and solution of TiF_4_ and NaF together with the irradiation of the Nd:YAG laser on enamel erosion/abrasion, Magalhaes et al^26^ determined that the TiF_4_ varnish could provide enamel protection against abrasion.

All of these investigations showed that CO_2_ laser irradiation together with fluoride is more effective in preventing caries than laser irradiation alone, even though Salazar et al^36^ found that caries has a great relation with the degree of enamel hardness. Thus, the studies mentioned above confirm the results of the present study.

Although many studies have shown the protective effect of CO_2_ laser in preventing the progression of caries,^8,11,19,38^ the results of the present study did not show any significant effects of CO_2_ laser (10.6 µm, 2 W power, for 10 s) on enamel hardness. However, it had significant effects when assisted by NaF and TiF_4_. It seems that this difference is related to laser parameters and the manner of its usage or the method by which the laser effect was measured. Laser parameters, such as pulsed or continuous wave, determine the amount of irradiation, and these are important factors in chemical changes (low temperature) or morphological changes (high temperature) on dental surfaces, and can also harm the pulp^38^ (the latter was not addressed in this research). In this study, no temperature rise was noticed. Temperature increase is greater in continuous wave than in pulsed mode. Therefore, it is recommended that the continuous and pulsed modes of laser irradiation and their effects on the pulp be compared in future studies.

## Conclusion

The current study corroborates that NaF varnish is not statistically significantly effective in increasing enamel hardness, while TiF_4_ in the form of varnish is a better option. Furthermore, although CO_2_ laser irradiation does not demonstrate a noticeable increase in enamel hardness, if laser is applied simultaneously with fluoride, it can increase the efficacy of fluoride. TiF_4_ varnish in conjunction with laser irradiation has proved to be more effective in increasing enamel surface hardness than the other treatments examined.
